# How a problem-based learning approach could help Japanese primary care physicians: a qualitative study

**DOI:** 10.5116/ijme.5de7.99c7

**Published:** 2019-12-26

**Authors:** Masayasu Seki, Yasuki Fujinuma, Masato Matsushima, Tatsuhiro Joki, Hideo Okonogi, Yasuhiko Miura, Iwao Ohno

**Affiliations:** 1 Division of General Medicine, Department of Internal Medicine, The Jikei University School of Medicine, Tokyo, Japan; 2 Centre for Family Medicine Development, Japanese Health and Welfare Co-operative Federation, Tokyo, Japan; 3 Division of Clinical Epidemiology, The Jikei University School of Medicine, Tokyo, Japan

**Keywords:** Continuing professional development, general practitioner, primary care, problem-based learning, focus group, Japan

## Abstract

**Objectives:**

This study aimed to identify training needs among primary care physicians in Japan who had no formal primary care training.

**Methods:**

We conducted a focus group interview with seven Japanese primary care physicians who had not previously undergone specialist training in primary care and had been recruited to a family medicine training program that used a problem-based learning approach. At the start of the program, the physicians attended the interview. The discussion was recorded, and the transcribed interview was analyzed using the Steps for Coding and Theorization method.

**Results:**

Three main themes emerged. First, there is a lack of standard re-education programs for physicians who move away from their specializations into primary care. Second, there is insufficient training on primary care in undergraduate and postgraduate medical education in Japan. Third, continuing professional development programs should cover the communication skills, attitudes, and behaviors necessary for primary care practice.

**Conclusions:**

This study clarified the needs to be addressed in our training program for primary care physicians involved in retraining in primary care. It is important to consider how to best include the communication skills, attitudes, and behaviors necessary for primary care among the topics covered in the program. As the program undergoes further iteration, it will be important to check whether it meets the needs of primary care practitioners. It will be necessary to investigate the needs of re-education programs for more physicians in many areas, and to emphasize the importance of primary care re-education in these abilities in undergraduate and postgraduate medical education.

## Introduction

Physicians must maintain their skills by undertaking continuous education or training to ensure that their professional practice remains appropriate throughout their working lives. Continuing professional development (CPD) plays an important role in the health care system and especially in quality assurance.^1^ CPD is clearly necessary for primary care physicians and general practitioners (GPs), who need training on how to manage the various reasons for physician–patient encounters and health problems.^2^

Generally, primary care is described as first-contact, accessible, continuous, comprehensive, and coordinated care.^3^ General practice is a phrase often used loosely to include GPs, primary care physicians, and family physicians. GPs are the only clinicians who work across all nine levels of care: prevention, pre-symptomatic detection of disease, early diagnosis, diagnosis of established disease, management of disease, management of disease complications, rehabilitation, palliative care, and counseling.^3^ The definition of core competencies and characteristics of general practice/family medicine describes 11 essential characteristics of the discipline that translate into six core competencies.^4^^,^^5^

Japan’s health care system is characterized by its universal insurance scheme, which gives enrollees the freedom to choose and purchase high-quality health care services from any facility at a relatively low cost.^6^ Japan continues to face many health-related challenges, such as an ageing population, low fertility rates, negative population growth, a stagnating economy, increasing unemployment, and the increasing burden from non-communicable diseases associated with the ageing population.^6^ Tackling these rising costs in the face of a growing population of older people will require drastic reforms in Japan’s health care systems and particularly its primary care systems.^6^

In Japan, the role of primary care physicians remains ambiguous. The policy vision lacks the clarity found for GPs in the United Kingdom or general internists and family practitioners in the United States.^7^ The Japan Primary Care Association was founded in 2010 and is now responsible for board certification of primary care and family physicians.^8^ A third-party organization that is distinct from The Japan Primary Care Association for managing the certification of Japanese GPs was established in 2017, and board-certified GPs are recognized as a new category of specialists under a board certification program beginning in 2018.^9^ Under this program, the concept of the GP in Japan is defined as combining that of the family practitioner, who specializes in outpatient care as the regular primary care physician in a clinic, with that of the hospitalist, who is mainly engaged in providing inpatient care at a hospital.^8^ Currently, about one-third of Japanese physicians establish their own private clinics and take on a primary care role after receiving 5–10 years of specialist training on specific organ systems at university hospitals or major medical facilities.^10^ However, many of these physicians are self-trained practitioners who do not necessarily receive systematic training in primary care to inform their primary care role. These physicians often find it difficult to access educational opportunities to obtain the necessary knowledge and skills for primary care while also managing their clinical responsibilities.^10^^,^^11^ Many Japanese private practitioners run a solo practice, so they play numerous roles in their community and have a substantial workload. This makes it difficult for them to dedicate time to education and monitoring at venues located far from their workplaces.^7^

There have been several studies of appropriate CPD programs. One study found that health care professionals preferred traditional lecture-based CPD activities but recognized that interactive sessions were more effective, helping them to retain information and change behaviors.^12^ Another review found that a problem-based learning approach could enhance physicians’ performance or improve health outcomes, but noted that there was limited evidence.^13^ A review of the literature on continuing medical education/CPD programs for GPs in rural areas found that it was not clear whether these programs improved physician performance and patient care.^14^

We have designed a new Family Medicine Brush-up Program, which is an interactive CPD program on primary care aimed at primary care physicians, using a problem-based learning approach. This program is designed to allow participants to learn family medicine through collaborative learning, acquiring the skills needed to practice as a primary care physician and handle the issues that they encounter in their workplaces (Appendix 1). The program is intended for physicians who did not formally train as family practitioners and who have approximately ten years of clinical experience in primary care. We worked with family physicians to create scenarios that reflect real medical situations in the primary care setting. Program participants identify the challenges posed by these scenarios and discuss how they should be approached.

It was, however, unclear whether this program would meet the needs of program participants. The purpose of this research was, therefore to clarify participants’ need for training, to inform the program content. A qualitative approach was chosen, because this allows the exploration of “real life” behavior and focuses on answering the questions “why” and “how”.^15^ Qualitative methods including interviews provide a deeper understanding of social phenomena than can be obtained from quantitative methods including questionnaires. 16 Interviews are appropriate in situations where little is known about the study phenomenon or where detailed insights are required from individual participants.^16^ We conducted a focus group interview before the start of the program to clarify the types of difficulties participants had experienced when learning about primary care through clinical practice. The participants were primary care physicians enrolled in the program who had not received specialist training in primary care. The ultimate objective was to use the results from the interview to improve future iterations of the program. We aimed to determine the needs to be addressed in the program by eliminating the gap between practice and training. CPD is thought to be most effective when there are clear needs and rationales for specific activities, when learning is structured to address these needs and rationales, and when follow-up CPD is provided to complete the training.^1^

## Methods

### Study design and participants

At the start of the Family Medicine Brush-up Program, a single focus group interview was conducted with program participants to determine the needs to address in the program.

We aimed to recruit 10 participants for the Family Medicine Brush-up Program, targeting Japanese physicians who had graduated at least 10 years earlier and who were practicing or had plans to practice primary care in the community. Participants had not undergone specialist training in family medicine. A total of 10 physicians applied for the program within the due date. Four of the authors (MS, YF, MM and IO) enrolled nine participants from the ten applicants, screening their documents to establish their motivation for participation and agreement to the use of a problem-based learning approach through the program. The focus group interview took place at the start of the program, so involved the seven physicians (described as A–G in Table 2) who attended that course. Two enrolled physicians who could not attend because of unforeseen circumstances and were not interviewed. These two absent physicians had a similar background in daily practice, so we considered that we were likely to achieve thematic saturation with seven participants. The participants met for the first time in the interview; they were unaccustomed to interviews and discussion on topics such as problem-based learning. This study was approved by the Institutional Review Board of the Jikei University School of Medicine (Study number: 27-277[8162]). All participants provided written informed consent to participate in this study. The research was conducted in accordance with the STROBE guidelines (Appendix 2).^17^

**Table 1:** Steps of Coding and Theorization and progression from raw focus group interview data to themes

Step	Description	Examples
Step 0	Raw interview data	“[…] In teaching residents, even though we took one course on medical interview techniques, I didn’t have the words to describe things. When I finally understood what family medicine was all about, I suddenly became aware that there is a framework to conceptualize and verbalize everything, and it came as a real surprise.”
Step 1	Noteworthy words or phrases from the text	medical interview, the words to describe things, aware, family medicine, conceptualize and verbalize
Step 2	Paraphrasing of the words and phrases in Step 1	Awareness of the conceptualization and verbalization of family medicine
Step 3	Concepts drawn from the text in Step 2	Experiences of difficulty with learning, the core competency of family medicine, problems with undergraduate and postgraduate medical education and continuing professional development
Step 4	Themes and constructs, with consideration of the context	The gap between practice and training

### Data collection

The participants received an explanation of the taped focus group interview process and gave their consent to participate. The focus group interview was conducted using the following guiding questions: 1) “Based on your experience to date when practicing or planning to practise primary care or family medicine, could you describe examples where your clinical practice went well or examples of your plans for clinical practice?”; 2) “Alternatively, could you describe examples where your clinical practice did not go well?”; and 3) “How did you resolve any difficulties?” The interview was audio-recorded using a digital recorder, with the written informed consent of all participants.

The interview was conducted in a quiet conference room of an external training facility outside the hospital where the authors and participants work. Three of the authors (MS, YM and MM) were in charge of the interviews. MS is a primary care physician and had little experience as the main interviewer. YM is a primary care physician and clinical ethicist and had experience as the main interviewer. MM is a primary care physician and clinical epidemiologist. YM was therefore the main interviewer, and MS and MM assisted. The researchers did not have any previous connection with the participants.

The interview was scheduled for approximately 60 minutes. In practice, the interview took 76 minutes, when the interviewers agreed that they had achieved theoretical saturation with no new comments from the participants.

**Table 2:** Characteristics of the study participants

Group	Sex, Age	Practice setting	Previous specialization
A	M, 40s	Private clinic	Reconstructive surgery
B	M, 40s	Private clinic	General practice, emergency medicine
C	M, 30s	City general hospital	Connective tissue disease
D	F, 30s	City general hospital	Internal medicine
E	F, 30s	Private clinic	General practice, primary care
F	F, 40s	University hospital	General practice, primary care
G	M, 40s	City general hospital	Internal medicine

### Data analysis

We analyzed the content of the focus group interview using the Steps for Coding and Theorization (SCAT) method.^18^ SCAT is a method of analysis that segments word-based data, such as observation records or interview records, and devises and appends codes following the four steps described in Table 1. This analytical method describes the storyline and theory by drawing out the themes and constitutive concepts. This method is also useful for analyzing qualitative data from relatively small samples, such as a single case or a case-free description on a questionnaire. This method was therefore considered appropriate for this study, drawing on data from a single focus group interview with seven participants.

The SCAT method has four main steps. In the first, the text data were divided into small units and classified as meanings or ideas. In the second, each of these small units was labelled with an interpretive description.^19^ Using the verbatim transcript, two authors (MS and TJ) independently coded the text for SCAT Steps 1 and 2. Where opinions varied about how to paraphrase the text, the two researchers discussed the issue and agreed the interpretation.

Coding for SCAT Step 3 was set with “experiences of difficulty with learning”, “the core competencies of family medicine”, and “problems with undergraduate and postgraduate medical education and CPD” as the categorized constructs. These drew on previous research about the reasons for choosing to become a GP, and gaps between GP training and subsequent clinical practice.^20^^,^^21^^,^^22^^,^^23^ Three authors (MS, TJ, and HO) independently conducted the coding for SCAT Step 4. For themes and constructs where the assigned categories did not match, the three researchers discussed the issue and agreed on the final category allocation.

## Results

The research participants’ statements were divided into three categories: “no standard re-education program for primary care physicians to respond to changes in the clinical and practice setting”, “problems with undergraduate and postgraduate medical education on primary care”, and “content of CPD on primary care” (Table 3). This section provides extracts from the focus group interview illustrating this categorization of participants’ statements.

**Table 3:** Themes and constructs on program needs and primary care learning extracted from focus group interview

Themes and constructs	Contexts
No standard re-education program for primary care physicians to respond to changes in the clinical and practice setting	Career change: surgeon, clinic inheritance, role in the workplace
Problems with undergraduate and postgraduate medical education on primary care	
Content of continuing professional development on primary care	Communication skills, credentials of primary care physicians, contrast with specialists on specific organs

No standard re-education program for primary care physicians to respond to changes in the clinical and practice setting:

“I struggled with whether I could continue as a surgeon for the next 20 or 30 years.” (Male, 40s, private clinic, reconstructive surgery)

“I knew that there were no educational or training programs that supported becoming a private practitioner or inheriting a private clinic.” (Female, 30s, private clinic, general practice and primary care)

“As one gets older, it is only natural that people start talking about the importance of working as a supervisor, and if you become a department director, obviously you start thinking about what that means outside the hospital as well as in the hospital.” (Male, 30s, city general hospital, connective tissue disease)

“I had been working in community health care, but when I returned after some time, I realized there were no tools for education. Rather than just turning my back on this issue, I feel that you need the tools and the right words.” (Female, 40s, university hospital, general practice and primary care)

Problems with undergraduate and postgraduate medical education on primary care:

“Japanese medical faculties are basically oriented toward specific specializations. I studied in an environment where the context of diagnosis and treatment was almost all within clinical practice.” (Male, 30s, city general hospital, connective tissue disease)

### Content of CPD on primary care

“Like empathy, communication requires certain skills, so I think they can be taught. I think that issues like whether you fit with a particular person’s character or policies are something else entirely. Training should teach doctors the communication skills they need for their work.” (Male, 40s, private clinic, general practice and emergency medicine)

“People around me asked whether I was really OK with not having specialization and not working together as a team on a specific career path in a university hospital.” (Female, 30s, private clinic, general practice and primary care)

“I think there are no specific characteristics or credentials for GPs or family practitioners. If specialists have conflicts with patients and their families about medical care, they are able to develop suitable characteristics and credentials through reflection on their own behavior.” (Female, 30s, private clinic, general practice and primary care)

## Discussion

Our study investigated training needs to inform the newly-developed Family Medicine Brush-up Program for the re-education of primary care physicians. It also explored problems with undergraduate and postgraduate medical education in primary care.

First, some statements made by the research participants suggested that there is no standard re-education program for Japanese primary care physicians who start as surgeons, become private practitioners, and then move into the role of manager or educator. A previous study found that physicians preferred a goal-oriented, part-time retraining program and wished to practice their specialty while retraining. 24 The same study reported that the most likely candidates for retraining were subspecialty physicians who currently provided some primary care.^24^ Specialists who are engaged in primary care and feel the need to learn about primary care seem to be appropriate participants for our program. This program, therefore, seems to meet the needs of participants who expect re-education.

Some programs using a problem-based learning approach have contributed to improved knowledge on disease management and better critical appraisal skills, as well as improved self-reported confidence and self-efficacy in managing the disease. These programs tend to improve physician performance.^13^^,^^25^ They may, therefore, be useful for primary care physicians who need to learn about the treatment of specific diseases. However, it is unclear whether the core competencies and characteristics of family medicine and primary care can be learned through a program using a problem-based learning approach.^5^ The challenge faced by our program is how to incorporate the core competencies and characteristics into a care-based learning approach teaching about the diseases that primary care physicians must learn to treat.^5^

Second, our study participants’ statements suggested that the Japanese system of undergraduate education and specialist medical training mainly focuses on the biomedical aspects of diagnosis and treatment. In Japan, one-third of medical specialists who have practiced their specialty for some time are involved in primary care as private practitioners.^10^ We therefore believe that undergraduate education and specialist medical training should include instruction in skills necessary for providing primary care, such as training in family medicine and on the temperament and communication skills needed to be a primary care practitioner. Japan must continue the debate over how its medical education system can be expanded, alongside the introduction of the board certification system for GPs.

Finally, some statements made by the research participants indicated that it is possible to teach the attitudes, communication skills, and professionalism needed to be a primary care practitioner. Previous research has suggested that the knowledge and skills required of GPs are fairly easily learned, but the challenge is how to learn the necessary attitudes and behaviors.^26^ In other studies, it might have been difficult to develop the required attitudes, behaviors, and communication skills through a problem-based learning approach supported by clinical scenarios, because the approach is primarily designed as a way to teach about diseases and conditions.^5^ When considered alongside statements highlighting the inadequacies of the Japanese system of undergraduate medical education and postgraduate specialist training, it is clear that our program and other retraining programs on the practice of primary care need to cover the attitudes and behaviors needed as a primary care practitioner.

The program described in this study could be described as an example of case-based learning because scenarios are used that reflect real primary care settings. Care-based learning is defined as a learning and teaching approach that aims to prepare students for clinical practice through the use of authentic clinical cases.^27^ These cases link theory to practice through the application of knowledge to the cases and encourage the use of inquiry-based learning methods. ^27^ Care-based learning promotes learning through the application of knowledge to clinical cases by students, enhancing the relevance of their learning and promoting their understanding of concepts.^27^ Further research may be needed to decide how our program can be adapted and improved.

### Limitations

The research participants in this study wanted to take part in the Family Medicine Brush-up Program, so this group may have been particularly interested and motivated to learn about primary care from the outset. It is possible that physicians with less interest or motivation to engage in this training would have different thoughts on this topic.

The focus group interview targeted physicians participating in the first iteration of this program, but two of the program participants were absent at the time of the interview. This study may therefore not have elicited all possible opinions about the difficulties of learning through the practice of primary care.

This study was based on a single focus group interview. It is unknown whether similar results would be obtained using multiple group interviews. We should consider increasing the number of program participants and conducting multiple focus group interviews in future.

The research participants were physicians who were not certified as family medicine specialists. However, numerous statements made during the focus group interview concerned the study of family medicine, suggesting that participants were learning about family medicine despite not having trained as specialists in this field. The focus group interview in this study might have produced different content if it had been based on discussions with physicians who were certified specialists or with physicians who knew nothing about family medicine studies. Besides, the authors were involved in the whole process of recruiting participants, creating guide questions, and conducting interviews. This may have helped to provide deeper discussions in the interview and enable full investigation into the needs of the participants. However, it might also have biased the researchers towards extracting comments about the needs they had previously identified for the program.

## Conclusions

This study clarified the needs to be addressed in our CPD program for primary care physicians involved in retraining in primary care. We must consider how best to include training in the communication skills, attitudes, and behaviors necessary for primary care among the topics covered in the program. It will be necessary to check whether further iterations of the program meet the needs of primary care practitioners. In further research, we will consider what program participants have learned and what changes were made in their practice as a result of this learning.

It will be necessary to investigate the needs of re-education programs for more physicians in primary care and other areas, and to emphasize the importance of primary care re-education focused on these abilities in undergraduate and postgraduate medical education.

### Acknowledgments

The Family Medicine Brush-up Program is funded by the Jikei University School of Medicine as part of a project entitled “Building General Practice Capability from Pre-graduate to Life-long Learning – for the Promotion of Clinical Research in the Community”. This work was supported by the

Japan Society for the Promotion of Science (JSPS) KAKENHI Grant-in-Aid for Young Scientists (B) Number 16K19179. We thank the interviewees for their time and participation. We thank Jennifer Barrett, PhD, and Melissa Leffler, MBA from Edanz Group (www.edanzediting.com/ac) for editing a draft of this manuscript and helping to draft the abstract.

### Conflicts of Interest

The authors declare that they have no conflicts of interest.

## Supplementary material

Supplementary file 1 Appendix 1. Competencies that participants can earn by taking the Family Medicine Brush-Up Program

Supplementary file 2 Appendix 2. STROBE Statement - checklist of items that should be included in reports of observational studies
