# Supplementary material for: How a problem-based learning approach could help Japanese primary care physicians: a qualitative study
Source: Int J Med Educ. 2019 Dec 26;10:232–40. doi: 10.5116/ijme.5de7.99c7 (PMC7246125; doi:10.5116/ijme.5de7.99c7)
Supplement: Supplementary file 1 — Appendix 1. Competencies that participants can earn by taking the Family Medicine Brush-Up Program [file ijme-10-232-S1.pdf]

Appendix 1.

Competencies that participants can earn by taking the Family Medicine Brush-Up Program

I. Management of typical health problems seen in outpatient primary care

Pediatric medicine – adult care – older people's care	Terminal care
Women's health	Rehabilitation
Mental health	Vaccination
Chinese medicine	First aid in clinic
Musculoskeletal, surgery, ophthalmology, otorhinolaryngology	

II. Understanding the principles of family medicine and clinical practice based on these principles

Patient-centered clinical method	Family-oriented primary care
Biopsychosocial model	Inter-professional work
Health promotion and prevention	Clinical ethics
Patient–doctor relationship, health care context and continuity	Behavior modification
Approach to complexity and uncertainty	Reflective learning

III. Communication and understanding of outpatient care organization

Communication and medical interview	Tests performed in the clinic
Clinical problem-solving	Evidence-based medicine
Guidelines on diagnosis and treatment	Professionalism
Approach to minorities and socially disadvantaged groups	Managing your medical facility
